# Tuning the Mesopore Network in Meso-Macroporous Silica Monoliths by Hydrothermal Treatment – A Physisorption Study

**DOI:** 10.1021/acs.langmuir.5c00572

**Published:** 2025-05-28

**Authors:** Usman Ali, Rafael Meinusch, Kevin Turke, Peter R. Schreiner, Bernd M. Smarsly

**Affiliations:** † Institute of Physical Chemistry, 9175 Justus Liebig University Giessen, Heinrich-Buff-Ring 17, 35392 Giessen, Germany; ‡ Center for Materials Research, Heinrich-Buff-Ring 16, 35392 Giessen, Germany; § Institute of Organic Chemistry, Justus Liebig University Giessen, Heinrich-Buff-Ring 17, 35392 Giessen, Germany

## Abstract

Macro-mesoporous silica monolith columns, prepared by a sol–gel procedure developed by K. Nakanishi, show beneficial flow and separation properties due to their 3D-interconnected macropores in combination with mesopores, providing a high surface area. Building on this, they are routinely used in analytical liquid chromatography. Within the synthetic process, fine-tuning of the mesopore dimension and interconnection is achieved by an etching step involving hydrothermal treatment under basic conditions, typically in the range of 80 °C–100 °C. The present study aims to unravel details of this harsh procedure by a comprehensive analysis of the resulting mesoporous network. Thus, a series of silica monoliths was prepared across a range of hydrothermal treatment temperatures (HTT) between 80 and 110 °C, thereby tuning the mesoporosity. Mercury intrusion porosimetry confirmed that enhanced HTT does not alter the macropore dimension and only affects the mesopore space. The study employed state-of-the-art physisorption analysis applying two adsorptives, Ar (87 K) and N_2_ (77 K), to identify changes in the mesopore size and network connectivity as a function of HTT. Also, advanced hysteresis scanning was performed on the same materials, providing independent insights into pore network effects. These analyses indicate that increasing HTT systematically enhances the average mesopore size from 8 nm (80 °C) to approximately 25 nm (110 °C) and widens the pore size distribution, pointing to pronounced dissolution of SiO_2_ at higher HTT. Surprisingly, the total mesopore volume remains constant upon increasing the HTT, implying a dissolution-reprecipitation mechanism for SiO_2_, rather than mere etching. Importantly, the in-depth porosity analysis reveals an increase in the size of necks, which reduces restrictions in the mesopore network connectivity. Furthermore, the data are in line with a recently proposed spatial mesopore size gradient in monoliths, which we find to be relevant at lower HTT and to systematically diminish toward higher HTT.

## Graphical abstract

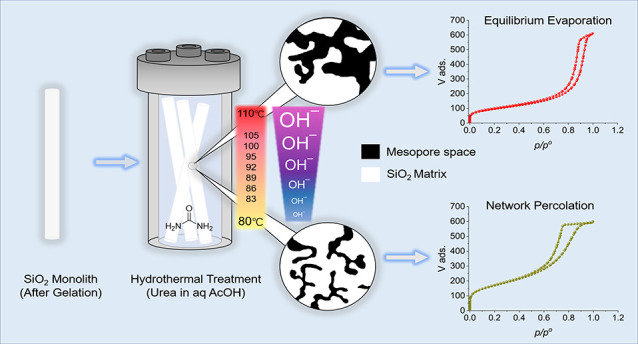

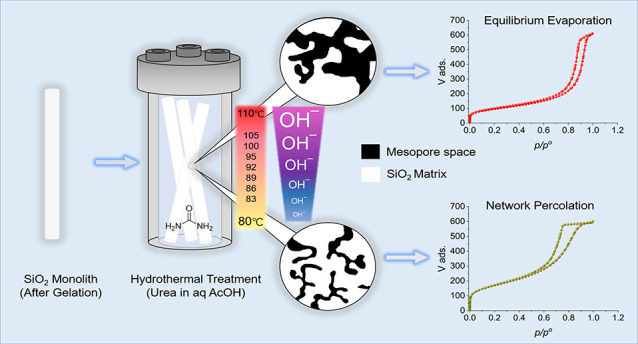

## Introduction

Complex meso-macroporous structures have become an important field of materials science in recent years, and major progress has been achieved for different materials such as silica, zeolites, carbons, ceramics, cellulose, clays, and metal–organic frameworks (MOFs), etc.^1–8^ The attention dedicated to meso-macroporous materials is certainly spurred by their commercialized applications in the fields of adsorption, separation science, and catalysis. Still, researchers continuously succeed in elucidating and overcoming limitations related to diffusion, accessibility, and mass transport of these materials through analytical and synthetic methods.^9–11^ These properties can be improved by finely modifying the macro- and mesoporous network.^12–18^

Hierarchical silica monoliths containing both meso- and macroporosity have emerged as particularly suitable materials due to their enhanced mass transfer properties, large surface area, well-defined macroporous skeleton, and standardized sol–gel-type synthesis based on readily available starting compounds.^12,19,20^ The special type of silica monoliths introduced by Nakanishi et al., possessing tunable meso- and macropore sizes and high surface area, are highly useful for various flow applications in heterogeneous catalysis, separation or adsorption, high-performance liquid chromatography (HPLC), super thermal insulators, and applications requiring self-standing bodies.^20–27^ They are particularly advantageous with regard to uniform hydraulic flow and access to active sites compared to other porous materials used for similar applications, such as organic polymer monoliths and particle-based fixed beds.^28–30^ Specifically, SiO_2_ monoliths potentially allow for optimizing the mobile phase velocity (contact time), reaction and adsorption selectivity, mass and heat transfer (efficiency), and specific surface area (loading capacity) in a distinct fashion.^13,31^ Owing to these features, these materials are commercially used in HPLC as a stationary phase, typically clad with steel or a specific polymer.^32–35^

However, adapting these hierarchically porous silica monoliths to specific needs still faces fundamental challenges related to the synthesis: aside from the macropore space, potential problems may arise due to (I) the presence of micropores and (II) potential restrictions in mesopore connectivity.^29,30,34^ These two phenomena might lead to potentially poor connections between and access to the mesopores, causing, e.g., pore blocking and potentially resulting in limitations for certain liquid-phase applications. Such hindered connectivity (a) limits the accessibility of guest species to the active sites, (b) causes poor diffusion and mass transfer, and (c) creates steric limitations for larger organic molecules, which are relevant issues, especially in organocatalysis.^36–39^ To overcome these limitations, an in-depth understanding of the pore-generating synthetic steps is required, thus enabling fine-tuning of mesopore size and connectivity.

Hence, in the following, a brief description of the synthesis of meso-macroporous SiO_2_ monoliths, based on the “Nakanishi process”, is given. The macropore network originates from phase separation during hydrolysis and condensation reactions of silicon alkoxides and the simultaneous induction of phase separation by spinodal decomposition of an organic polymer in an acidic environment. The next step involves hydrothermal treatment in the presence of urea at elevated temperatures (in the range of 80 °C and above), resulting in a pH increase, which, in turn, causes dissolution and reprecipitation of SiO_2_. For instance, hydrothermal treatment is typically performed at 95 °C, yielding an average mesopore diameter of approximately 13 nm, which is typically used in the HPLC application of these monoliths.^23^ This hydrothermal treatment widens pristine micropores into mesopores and thus determines the final mesopore space with regard to the mesopore size distribution and their mutual connection. The wide use of this route is based on its straightforward synthetic steps that lead to a specific meso- and macropore structure and surface area, with high reproducibility.^40^ By controlling different parameters during synthesis, one can tune the meso- and macropores at different steps of synthesis as per application demands.^41,42^

While the hydrothermal treatment has been extensively studied for its impact on the final transport properties, the mechanistic details of structural and chemical changes, their impact on the final mesopore size distribution, and the connectivity of mesopores are still a matter of research, especially regarding their dependence on the treatment temperature. In previous studies, the temperature applied during the hydrothermal treatment was quite high, but covering a broader temperature range is expected to provide additional insights.^43–50^

Thus, to further elucidate the generation of mesoporosity and the influence of hydrothermal treatment on the connectivity of mesopores, we studied silica monoliths treated over a range of HTTs between 80 and 110 °C.

The advancements in state-of-the-art physisorption analysis and the interpretation of data are mainly based on density functional theory (DFT), allowing for the evaluation of consistent and meaningful pore size distributions (PSDs) over a broad range of micro- and mesopores. For advanced pore network analysis, we performed physisorption studies of the samples using two different adsorptives, argon (87 K) and N_2_ (77 K), and compared the PSD data to interpret pore networking effects (pore blocking, cavitation, or independent pore networks) in silica monoliths. This analysis applied the methodology for pore network analysis first reported in 2006 by Thommes et al.^51^ The use of both nitrogen and argon is essential for accurate pore network analysis due to their differing thermodynamic states and adsorption behaviors. Using nitrogen (77 K) for pore analysis has limitations, especially for micropores, due to its specific interactions with surface groups that affect pore filling pressure and diffusion. In contrast, physisorption using argon at 87 K is more suitable- for several reasons. By comparing desorption pore size distributions obtained with both gases, one can distinguish between pore blocking and cavitation mechanisms, providing a clearer understanding of pore connectivity and behavior.^52^

Hysteresis scanning is another advancement in physisorption science that reveals the correlation between the pore network, its connectivity, pore shape, and PSD. In hysteresis scans, the variation in gas pressure during the process of adsorption and desorption generates partial filling and emptying of pores in the range of relative pressure where hysteresis occurs. This leads to hysteresis lines originating in the region of the hysteresis envelope, and their shape provides insight into the connectivity of the mesopores, for instance, in terms of an equilibrium evaporation transition, network percolation (pore blocking), or cavitation. In the past few years, a lot of work has been devoted to scanning hysteresis lines starting from the desorption branch, but far fewer studies address the scanning of adsorption isotherms.^53–57^ In 2012, Cimino et al. worked on studying the scanning of adsorption and desorption isotherms of materials possessing different kinds of pore networks. They introduced percolation models and partial correlation models for network effects in mesoporous materials. They also developed a computational method for calculating the distribution of pore and neck sizes as well as the network coordination number.^54^ Svidrytski et al. studied the hysteresis scans of both adsorption and desorption isotherms using different kinds of mesoporous silica materials spanning ordered (SBA-15, KIT-6) to disordered (random silica with restricted mesopores) mesopore networks. Their work found good agreement between modeling and experimental isotherms for materials exhibiting type IV­(a) isotherms with a H1 hysteresis loop (for ordered mesopores) and a H2 hysteresis loop (for random silica).^55^ Thus, we used desorption hysteresis scanning experiments to elucidate fine differences in mesopore connectivity as a function of the HTTs.

In summary, variations in the HTT alter the rate of chemical changes during the process, which, in turn, might affect the evolution of the mesopore network. This study is an attempt to understand these changes by state-of-the-art physisorption analysis using an advanced pore network analysis approach over a range of samples treated at different HTT and thus to improve the overall understanding of the mesopore networks of this important class of silica monoliths. Moreover, mercury intrusion porosimetry and SEM analysis were carried out to identify possible changes in the macropore structure induced by elevated HTT.

## Materials and Methods

Tetramethoxysilane (TMOS, 98%) was purchased from Thermo Scientific, acetic acid (100%) from Carl Roth, urea from Sigma-Aldrich, and poly­(ethylene oxide) (PEO, *M*_n_ = 10 000 g/mol) from Fluka Analytical.

### Characterizations

*SEM*: A Smart SEM MERLIN (Carl Zeiss) scanning electron microscope was used for scanning electron microscopy with an acceleration voltage of 1 kV. Samples were sputtered with platinum for 60 s before analysis.

*Mercury Intrusion Porosimetry*: The morphology of the macropores was analyzed using a mercury porosimeter (Pascal 140/440, Thermo Fisher Scientific, Rodano, Italy) with a pressure range of 0–400 MPa. Washburn’s equation was used to analyze the data, supported by the instrument software (Sol.I.D). A surface tension of 0.48 N/m and a contact angle of 140° were assumed for the intruded mercury.

*Physisorption:* Physisorption studies using Ar (87 K) and N_2_ (77 K) were carried out using an automated gas adsorption station (Autosorb iQ2, Quantachrome Corp., Boynton Beach, FL). For the measurements, a small piece of each silica monolith sample (20 mg) was placed into a glass tube with a cylindrical end measuring 9 mm in diameter. Each sample was degassed at 120 °C for 12 h before starting the physisorption experiment. For Ar (87 K) physisorption measurements, the samples were kept at 87 K using liquid Ar. The surface areas were calculated by the Brunauer–Emmett–Teller (BET) model, while pore size distributions (PSD) were determined using the NLDFT-Kernel (Ar at 87 K, zeolite/silica cylindrical pores) applied to both the adsorption and desorption branches. The BET model approach was also used to calculate the surface areas for N_2_ (77 K) physisorption, while pore size distributions were determined using the NLDFT-Kernel (N_2_ at 77 K on silica cylindrical pores) for both the adsorption and desorption branches. Cumulative pore volume and cumulative surface area distributions were also calculated for comparison. All these calculations were performed by the ASiQwin software, version 5.21. To elucidate the porous structure in different samples, hysteresis scanning was measured for both the adsorption and desorption branches of all samples. For the hysteresis scanning, four data points were selected from the isotherms, which were then used for the desorption hysteresis scan.

### Synthesis of Silica Monoliths

A modified Nakanishi route was used to synthesize silica monoliths. 1.21 g of poly­(ethylene oxide) (PEO, *M*_n_ = 10 000 g/mol) and 0.9 g of urea were dissolved in 10 mL of 0.01 M acetic acid, and the solution was magnetically stirred at room temperature until all the particles dissolved in the aqueous acidic solution. The clear solution was then shifted to an ice bath for 20 min, after which 5.6 mL of tetramethyl orthosilicate (tetramethoxysilane or “TMOS”) was added and stirred for an additional 20 min at 0 °C. The ice bath was then removed, and the solution was stirred at room temperature for the final 10 min. For the next step, i.e., gelation, we used stainless-steel tubes with an inner diameter of 3.8 mm and a length of 15 cm. The tubes had already been immersed in a thermostat (Julabo F26, Julabo GmbH, Germany) set to maintain a precise constant temperature of 22.5 °C. The ends of the tubes were sealed with plastic caps wrapped with parafilm, and 1.2 mL of the solution was added into each tube using a syringe. After 20 h of gelation at 22.5 °C, the silica monoliths were removed from the stainless-steel tubes and transferred into centrifuge vessels containing 45 mL of an aqueous solution of urea and acetic acid for hydrothermal treatment (9 g of urea dissolved in 100 mL of 0.01 M acetic acid). See Figure 1 for the mechanism and a schematic illustration of the Nakanishi-type synthesis.

**Figure 1:**
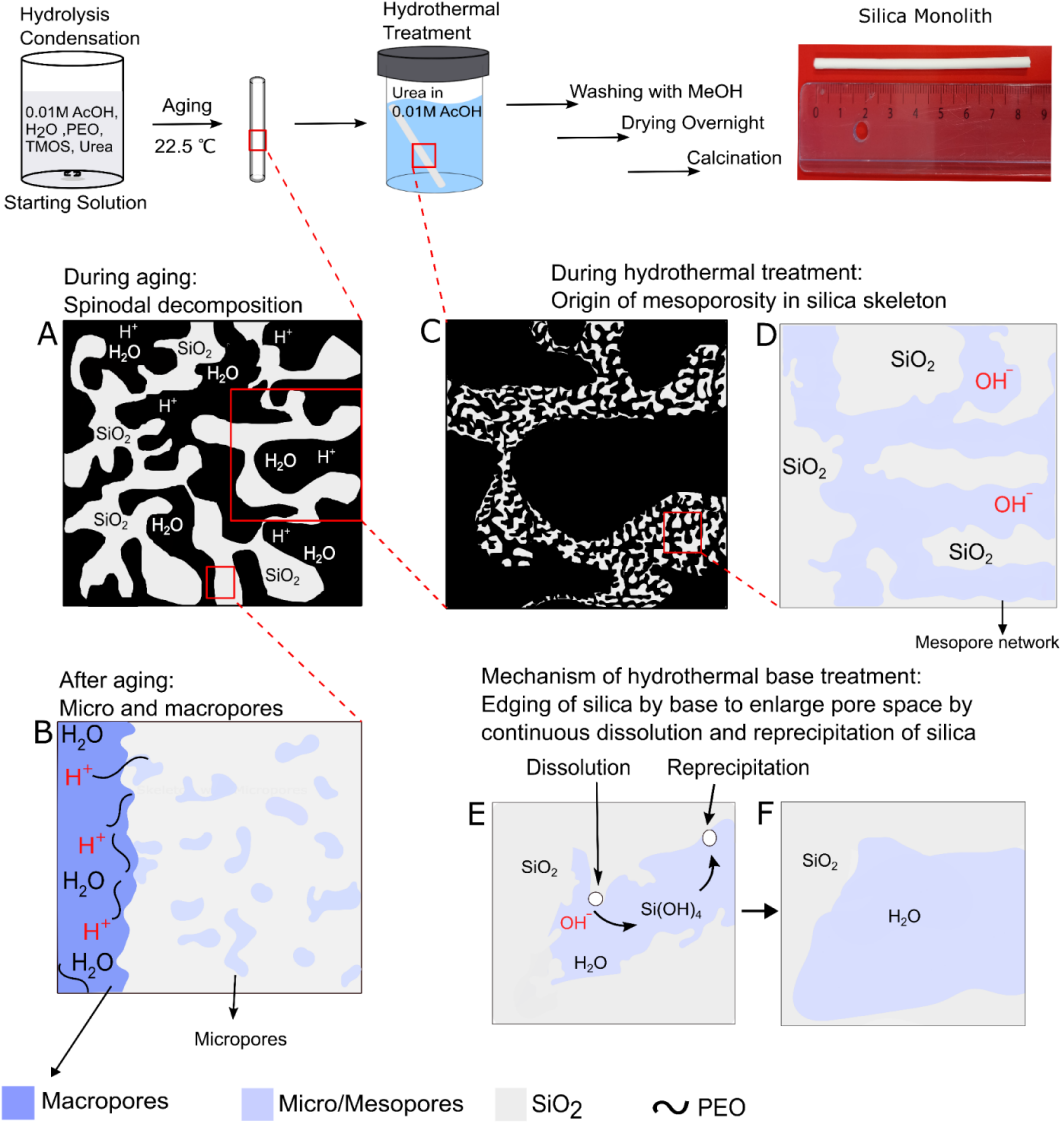
Schematic synthesis of silica monoliths by the “Nakanishi route”, and origin of porosity at different steps of synthesis: (A) origin of micro- and macropores during aging at 22.5 °C that separates the water-rich phase (macropores) and silica-rich phase after spinodal decomposition; (B) generation of micropores inside the silica skeleton during gelation, which enlarge into mesopores during hydrothermal treatment; (C, D) emergence of mesopores during hydrothermal treatment in basic pH that dissolves silica into soluble siliceous species and enlarges the micropores into a mesoporous network; dissolution (E) and reprecipitation (F) of silica during hydrothermal treatment.

### Hydrothermal Treatment Conditions

For hydrothermal treatment, the centrifuge vessels containing silica monoliths in 45 mL of solution (9 g of urea dissolved in 100 mL of 0.01 M acetic acid) were tightly closed, wrapped with parafilm, and then placed into an oven. An autoclave was used for those monoliths that were hydrothermally treated at temperatures of 100 °C and above. Table 1 shows the temperature and timing parameters used for the hydrothermal treatment of different samples. From 80 to 95 °C, the hydrothermal temperature was systematically increased in steps of 3 °C, and by 5 °C from 95 to 100 °C to match the boiling point of water, then to 105 and 110 °C. The temperature ramp and holding time for every hydrothermal treatment were kept at 10 h each, so the total time for each hydrothermal treatment was 20 h, except SiO_2_-100 for which a temperature ramp lasting 13 h and a holding time of 15 h were applied to observe the impact of increased hydrothermal treatment timing on PSD. The names of the samples reflect the temperature at which they were treated hydrothermally (for example, SiO_2_-80 was treated at a HTT of 80 °C).

**Table 1:** Parameters for the Hydrothermal Treatment of Silica Monoliths

Sample name	Hydrothermal treatment temperature (HTT) (°C)	Temperature ramp (h)	Holding time (h)
SiO_2_-80	80	10	10
SiO_2_-83	83	10	10
SiO_2_-86	86	10	10
SiO_2_-89	89	10	10
SiO_2_-92	92	10	10
SiO_2_-95	95	10	10
SiO_2_-100-1	100	10	10
SiO_2_-100	100	13	15
SiO_2_-105	105	10	10
SiO_2_-110	110	10	10

### Sensitivity of Synthetic Route for Silica Monoliths

The synthetic route for silica monoliths is highly sensitive to even the slightest change in any of the parameters, including the concentration of polymers, variations in pH, contamination, pressure changes, or, most importantly, slight changes in temperature and timing. Even a slight change in any parameter can lead to differences in the size, shape, and connectivity of pores in silica monoliths. To tailor and control the porosity, it is important to have profound insight into the individual steps involved in the generation of micro-, macro-, and mesopores.^58^

During gelation, chemical spinodal decomposition occurs that separates two coexisting phases, namely, a solvent-rich phase and a silica/polymer-rich phase. Spinodal decomposition is a kinetic process in which two phases grow simultaneously, accompanied by the condensation of silica oligomers. The shape and size of the water-rich phase control the size and shape of the final macropore network, while the topology and size of the silica-rich phase determine the topology and dimension of the silica monolith skeleton (Figure 1A,B). The rate of condensation is controlled by temperature, which ultimately governs the mean macropore size during the gelation step.^12,14,41,59,60^ Recently, Meinusch et al. published a study on the influence of temperature changes during the gelation step, concluding that even a slight increase or decrease in temperature by only 1.5 °C leads to changes in the macropore size by a factor of 2.^61^

### Hydrothermal Treatment

The gelation step is followed by hydrothermal treatment of the silica monoliths in a solution of urea and acetic acid. This step is responsible for tailoring mesopores inside the monolithic silica skeleton, using basic media generated by the decomposition of urea into aqueous ammonia at elevated temperatures (Figure 1C,D). The hydrothermal treatment causes the widening of micropores and also etching (dissolution) of silica from a silica-rich phase into small dissolved siliceous species (formally Si­(OH)_4_) creating micro- and mesoporosity, and concurrently involving reprecipitation at nearby concave surfaces (Figure 1E,F). The etching of micropores and the rate of dissolution and reprecipitation depend on the HTT, which can thus be used to control the mesopore size. The hydrothermal step might be viewed as a process similar to Ostwald ripening or coarsening, during which silica dissolution occurs at convex surfaces while reprecipitation occurs at concave surfaces, thereby decreasing the overall curvature on the mesoscale. Increasing the temperature during hydrothermal treatment results in the broadening of the pore size distribution as well as the enlargement of mesopores.^44,62–66^

After the hydrothermal treatment was completed, the silica monoliths were removed from the oven and allowed to cool to room temperature. They were then immersed in 45 mL of HPLC-grade methanol, ensuring complete submersion for solvent exchange. This process was repeated every 24 h for three consecutive days to facilitate thorough removal of residual species.

Following solvent exchange, the monoliths were dried overnight in a fume hood and subsequently subjected to calcination at 330 °C. The calcination process involved a controlled heating ramp from room temperature to 330 °C under air over 15 h, followed by a 10-h holding time to ensure complete decomposition of residual organic polymer (PEO). Predrying overnight and using a slow heating ramp prevent cracking in the monoliths by reducing capillary forces during the calcination process. After calcination, the monoliths were characterized in detail to assess their meso- and macroporous structures.

## Results and Discussion

The synthesis follows previously reported protocols and yields the typical meso-macroporous makeup, with the macropores forming a continuous network of micrometer-dimensioned pores, while the mesopores are located in the skeleton separating the macropores.^40,61^ SEM analysis (Figure 2A) reveals that at lower HTT (SiO_2_-80, SiO_2_-83) the silica skeleton exhibits a well-defined shape with a uniform diameter of the strands. The mesopores inside the skeleton are visible on the broken silica arms. As the temperature increases, (SiO_2_-95, SiO_2_-100, SiO_2_-110) the skeleton appears more irregularly spread, which is a result of heterogeneity in the redeposition of dissolved silica on the skeleton surface, enhanced by the elevated temperature in the autoclave during hydrothermal treatment. At high HTT, the rate of silica dissolution increases and enlarges the mesopore size by trimming silica under more basic conditions and then randomly redepositing it onto the outer surface. SEM images further show no visible effect on the size of macropores during hydrothermal treatment, which is confirmed by mercury intrusion analysis in Figure 2B indicating an almost identical macropore diameter for all samples. This finding is expected because of the uniform temperature conditions during the gelation step. Yet, a significant difference in mesopore volume is observed in the pore size distributions (Figure 2B), revealing that the mesopore volume increases with an increase in HTT. This difference is due to (i) mesopores smaller than 3.5 nm, which are nonintrudable by mercury, and (ii) pore blocking of large mesopores connected by smaller ones. These effects are present in samples SiO_2_-80, SiO_2_-83, and SiO_2_-95 confirmed by physisorption data (see below). By contrast, the larger, independent open mesopores in SiO_2_-100 and SiO_2_-110 do not exhibit such hindrance of intrusion. A prominent effect is observable in the diameter of silica monoliths (Figure 2C), which decreases with increasing treatment temperature. This decline is due to corrosion of silica from the outer surface of the monolith by the base, which dissolves silica into aqueous solution, and shrinkage caused by vapor pressure exerted on the monolith surface. However, it remains difficult to determine which of these effects is the dominant contributor.

**Figure 2:**
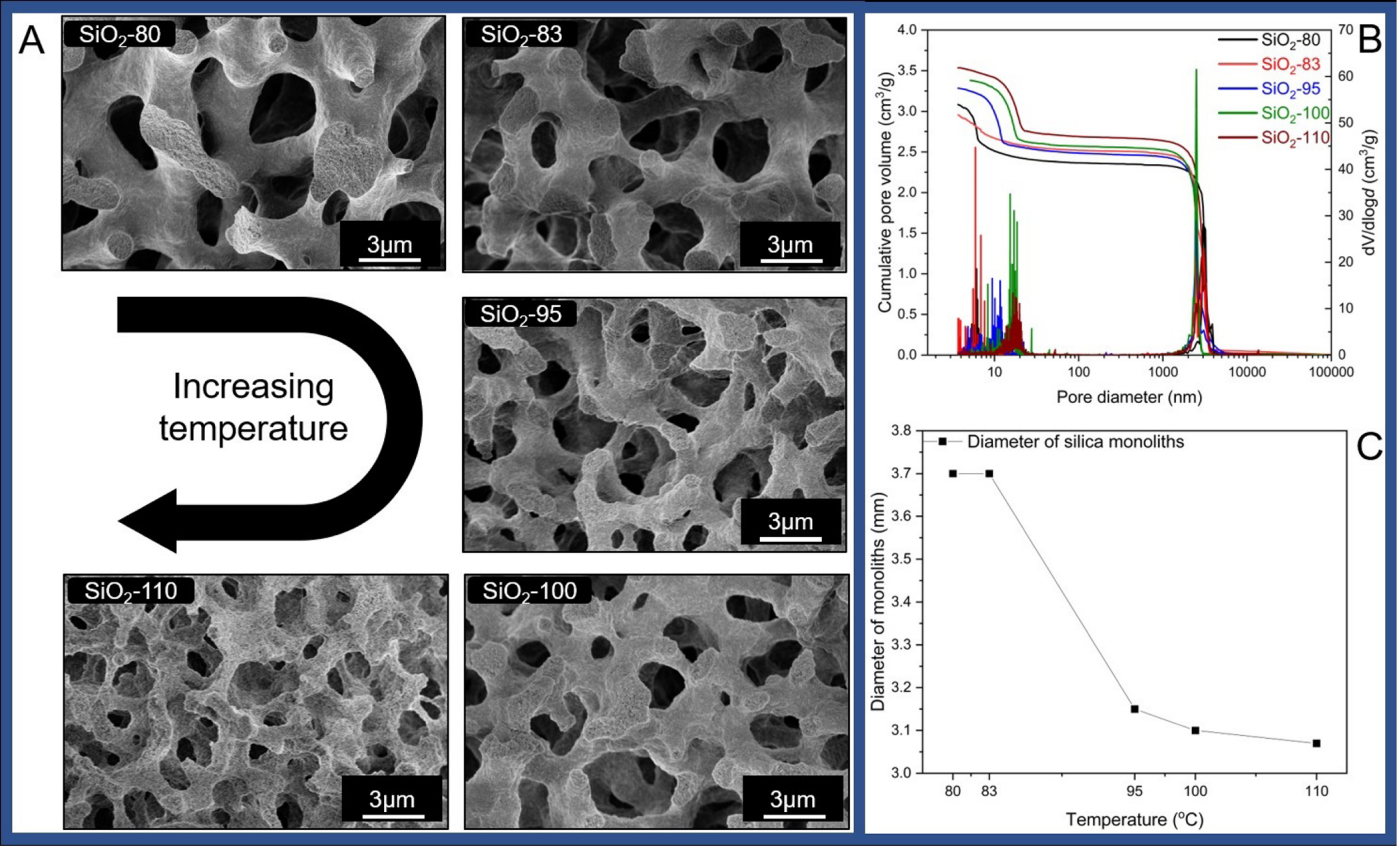
SEM images (A) of silica monoliths prepared at different HTT. The name of the samples indicates the HTT. An identical magnification was used for the SEM micrographs to ensure comparability of the skeleton morphology. With increasing HTT, it can be seen that the skeleton of silica monoliths become more irregular in shape due to strong etching effects. Furthermore, with increasing temperature silica reprecipitates on the outer surface rendering the skeleton surface coarser in appearance. (B) Mercury intrusion porosimetry data for silica monoliths showing good agreement in macropore sizes among all samples, which is due to the identical gelation temperature during the course of synthesis of monoliths that controls the size of macropores. The diameter of silica monoliths (C) gradually decreases as result of corrosion of silica with base from the outer surface and shrinkage due to vapor pressure exerted on outer surface during high HTTs.

To elucidate the influence of hydrothermal temperature on the mesoporous structure, especially with regard to mesopore connectivity, the monolithic silica rods were analyzed by Ar (87 K) and N_2_ (77 K) physisorption analysis.

Figure 3 reveals that all of the physisorption isotherms are of type IV­(a), while the hysteresis loops vary as the HTT increases. Taking SiO_2_-80 as a reference, the position of the condensation step shifts to higher relative pressures, implying that the mesopore size increases with an increase in HTT. Silica rods treated at lower HTT (SiO_2_-80 to SiO_2_-89) show a mixture of H2­(a) and H1 hysteresis loops (SiO_2_-83 and SiO_2_-89 isotherms in Figure S1), the latter being typical of silica monoliths that are well-known for their well-connected mesoporous network. A contribution of a H2­(a) hysteresis loop appears as a steeper step in the desorption branches and thus indicates hindered emptying from filled mesopores, caused by “pore blocking” or “cavitation”. In either case, the cavity remains filled until the neck evaporates at lower relative pressure *p*/*p* ^o^. We would like to note that the incline in the desorption branch can possibly be influenced additionally by the size distribution of mesopores and the necks connecting them. While the Ar (87 K) and N_2_ (77 K) isotherms of SiO_2_-80 to SiO_2_-89 exhibit a major contribution of a H2­(a) hysteresis loop, with higher HTT the shape of the isotherms shows continuously increasing contributions of H1 hysteresis, accompanied by an increase in mesopore size (see below). A more drastic change can be seen when silica rods are hydrothermally treated at 92 °C and above. For the highest HTTs (SiO_2_-100, SiO_2_-105, SiO_2_-110) almost ideal H1 hysteresis loops were observed as a result of well-connected and accessible mesopores. Such a systematic change in the shape of isotherms with moderate steps in the applied HTT offers the opportunity for fine-tuning mesopore diameters and connectivity. The differentiation between the different possible mesopore connectivity scenarios was performed on the basis of NLDFT-based pore-size distributions, see below.

**Figure 3:**
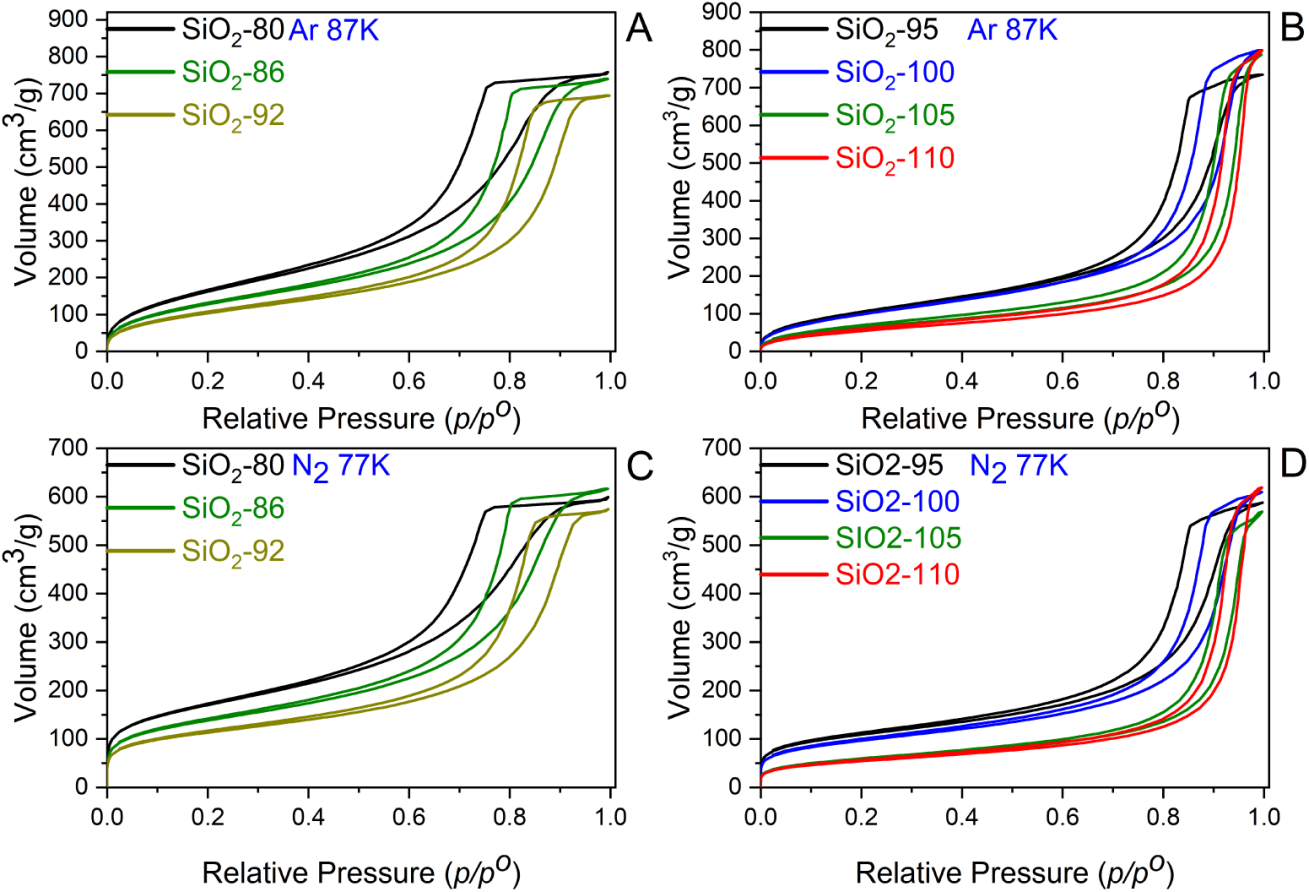
(A,B) Ar (87 K) and (C,D) N_2_ (77 K) physisorption isotherms of silica monoliths treated at different hydrothermal temperatures. The name of samples reflects the temperature at which they were treated hydrothermally (for example, SiO_2_-80 was treated at 80 °C). As the HTT increases, the condensation step shifts toward higher *p/p^o^* due to an increase in mesopore size, and the shape of isotherms changes from type H2 to mixture of H2 and H1, and finally to type H1 at higher temperatures. Only selected isotherms are shown here for better visualization. All of the isotherms of a total of nine samples can be seen in the Figure S1A–D.

### Influence of Hydrothermal Temperature Treatment on Cumulative Surface Area and Cumulative Pore Volume

Cumulative (Figure 4) and differential pore size distributions (PSDs) (Figure 6) were evaluated from physisorption data for Ar (87 K) and N_2_ (77 K) isotherms. The NLDFT cylindrical pore model was used to calculate the PSDs, which is known to provide accurate pore size distributions for silica materials of this type.^51,67^ For comparison, the NLDFT model of cylindrical and spherical pores was also applied to two samples. The results (Figure S2) showed negligible differences in PSDs with both models for SiO_2_-80 and SiO_2_-100 with argon; however, for nitrogen, where the NLDFT cylindrical-spherical kernel is only available for the desorption branch, the PSDs appeared low in volume but overestimated in size, which is not consistent with data from the cylindrical kernel of argon and nitrogen (Figure 5).

**Figure 4:**
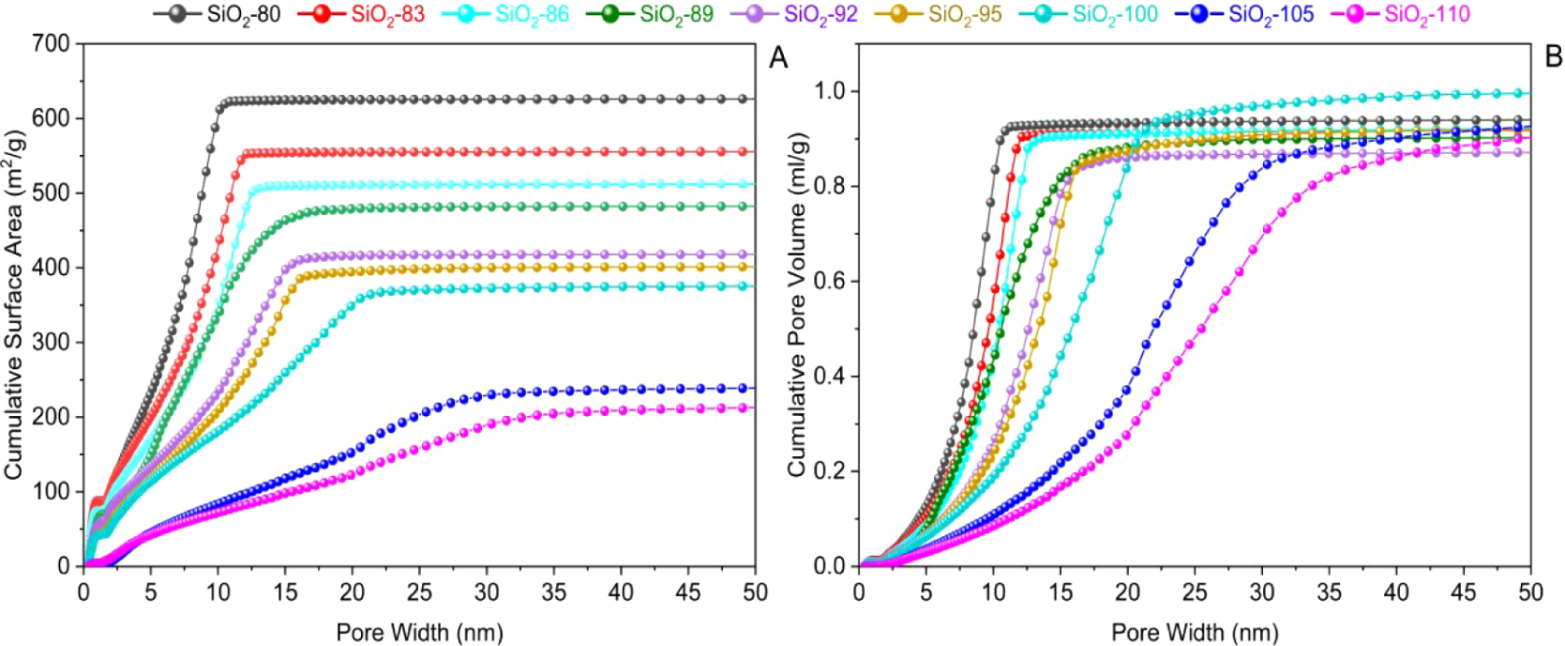
Cumulative surface area (A) and cumulative pore volume (B) obtained from desorption branches of Ar (87 K) physisorption isotherms by using an NLDFT cylindrical pore model.

**Figure 5:**
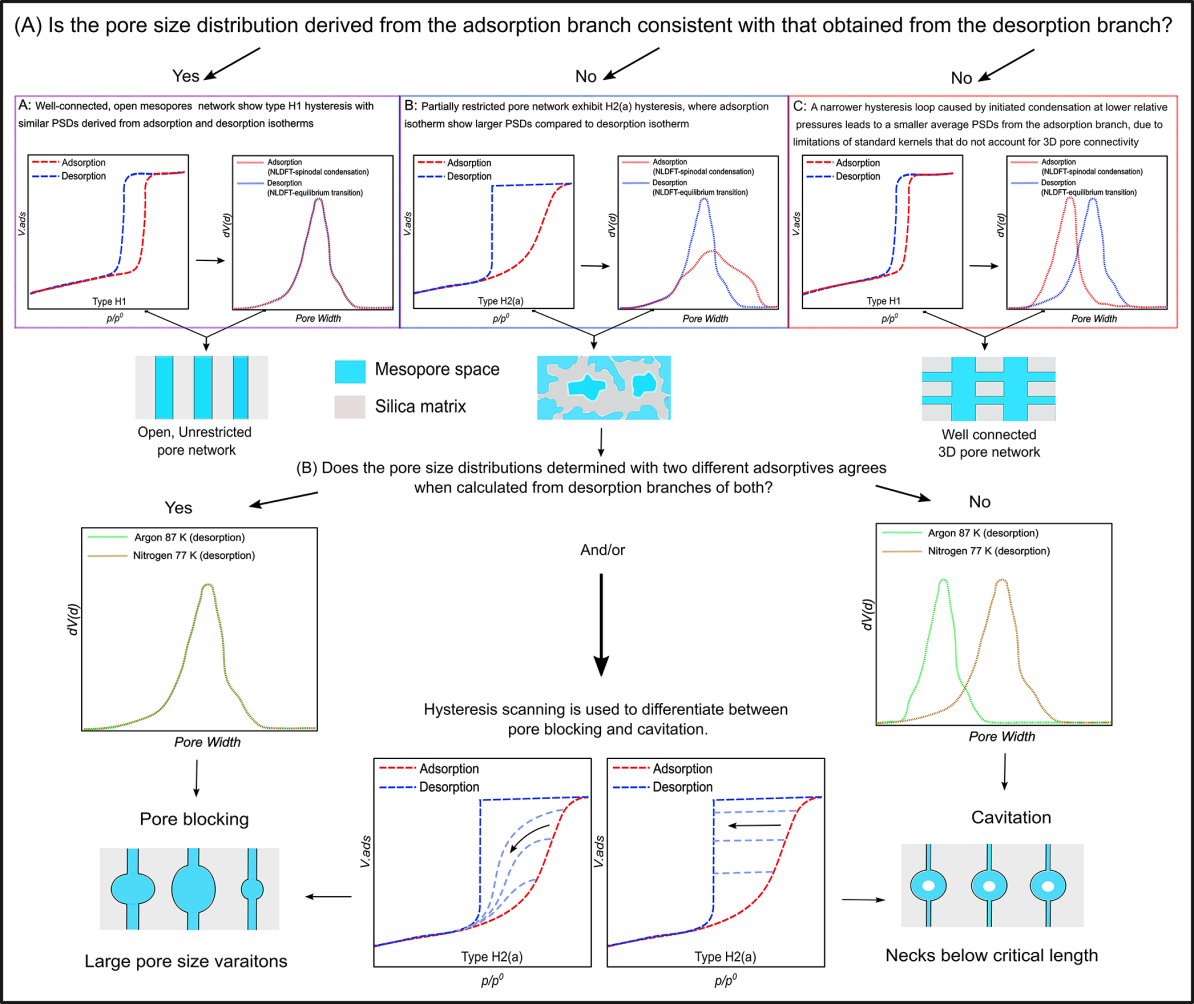
The advance pore network analysis approach used in this study (reported in detail by Schlumberger et al. 2021),^52^ where the use of two different adsorptives help to determine the pore network effects due to different thermodynamic behaviors of Ar (87 K) and N_2_ (77K in confined mesopore space. (A) Comparing pore size distributions from adsorption and desorption branches using NLDFT adsorption branch kernels and NLDFT equilibrium desorption branch kernels, respectively, to check for the presence of restricted pores. (B) If restricted pores are found, comparing results from different adsorptive gases can help to understand the desorption process. Differences in the pore size distributions from nitrogen (N_2_) and argon (Ar) desorption branches suggest cavitation. (C) A narrow H1-type hysteresis loop due to condensation starting at low relative pressure. This shifts the pore size distribution from the adsorption branch to smaller values because standard models (kernels) do not consider the 3D connectivity of pores. Desorption hysteresis scanning is also a useful method for probing pore network effects, where the shape and closure behavior of hysteresis branches help distinguish between pore blocking and cavitation mechanisms.

Thus, using the NLDFT kernel for cylindrical pores, the samples up to SiO_2_-100 showed a small micropore volume, which disappears upon increasing the HTT. Since the changes in the PSD are most prominent in the range of 5–20 nm, we conclude that micropores and small mesopores are converted into larger mesopores, confirming the general view on the impact of such hydrothermal exposure. Concomitantly, the overall surface area continuously decreases (Figure 4A and Table 2), from almost 560 m^2^/g (SiO_2_-80) to 350 m^2^/g (SiO_2_-100), and finally 200 m^2^/g (SiO_2_-110) upon enhancing the temperature in the hydrothermal treatment, as a result of small micro- and mesopores merging into larger mesopores. Quite interestingly, the overall cumulative pore volume is almost unaffected by varying the HTT (Figure 4B and Table 2), despite the marked change in the PSD. In the samples treated at hydrothermal temperatures between 80 and 90 °C with steps of 3 °C, the major contribution to the total pore volume results from mesopores in the range of 2 to 15 nm. Notably, increasing the hydrothermal treatment time for SiO_2_-100 from 20 to 28 h does not alter the PSD but leads to a slight increase in cumulative pore volume (Figure S3). For temperatures above 100 °C, mesopores larger than approximately 15 nm dominate the contribution to the mesopore volume (Figure 4B). Note that the gelation temperature and time were carefully kept uniform for the synthesis of all silica monoliths to avoid any effects of gelation conditions on mesoporosity, ensuring that the observed variations in PSDs can be attributed solely to the hydrothermal conditions. The influence of pores larger than 50 nm on cumulative surface area and cumulative pore size is shown in Figure S4. Hence, these systematic trends in the PSD, surface area, and pore volume indicate a widening and fusion of micropores and small mesopores upon dissolution of siliceous species, which do not remain in the solution but are deposited within the sample. It is well-established that an increase in hydrothermal temperature decomposes urea in aqueous solution into ammonia, raising the pH value. This increase in pH along with increased temperature, enhances the rate of dissolution of silica into Si­(OH)_4_ creating wider mesopores in monolithic rods.^68^ The constant overall cumulative pore volume also suggests that the rate of dissolution is similar to the rate of reprecipitation for all of the applied temperatures. From these physisorption experiments alone, however, it is impossible to conclude the whereabouts of this dissolved-deposited SiO_2_ material.

**Table 2:** Variation in Surface Area, Average Pore Size, and Pore Volume of Silica Rods Treated at Different Hydrothermal Temperatures, Determined by Ar Physisorption (87 K)

Sample	BET surface area (m^2^/g)	Average pore size (nm)	Total pore volume (mL/g)
SiO_2_-80	560	9	0.94
SiO_2_-83	480	11	0.92
SiO_2_-86	440	12	0.93
SiO_2_-89	420	13	0.93
SiO_2_-92	350	14	0.88
SiO_2_-95	360	15	0.93
SiO_2_-100-1	335	19	0.95
SiO_2_-100	350	19	ca. 1
SiO_2_-105	230	23	ca. 1
SiO_2_-110	200	27	ca. 1

## Mesopore Connectivity

For deeper insight into the progressive changes in the mesopore space, especially with regard to connectivity and dependence on the HTT, we calculated PSDs from both the desorption and adsorption branches of the isotherms (Figure 3).^69,70^ Following the advanced pore network analysis approach developed by Thommes et al.^5,51,52^ (Figure 5 ), network effects, and particularly possible pore emptying hindrance in the form of “pore blocking” or “cavitation”, can be identified and distinguished from one another by comparing PSDs determined from the adsorption a­(Figure 5, case C)­nd desorption branches in cases where pronounced hysteresis is observed. This analysis is based on the isotherms of two different adsorptives, namely Ar (87 K) and N_2_ (77 K). These two adsorptives have different thermodynamic states inside the mesopores, enabling a straightforward procedure for identifying possibly restricted pore emptying from the state of liquid fluid based on the desorption-based PSDs: If the PSDs obtained for Ar (87 K) and N_2_ (77 K) measurements are identical, then pore blocking is the dominant phenomenon. On the other hand, if the PSDs evaluated from Ar (87 K) isotherms are shifted compared to the N_2_ (77 K) analysis, then desorption is governed by cavitation.

**Figure 6:**
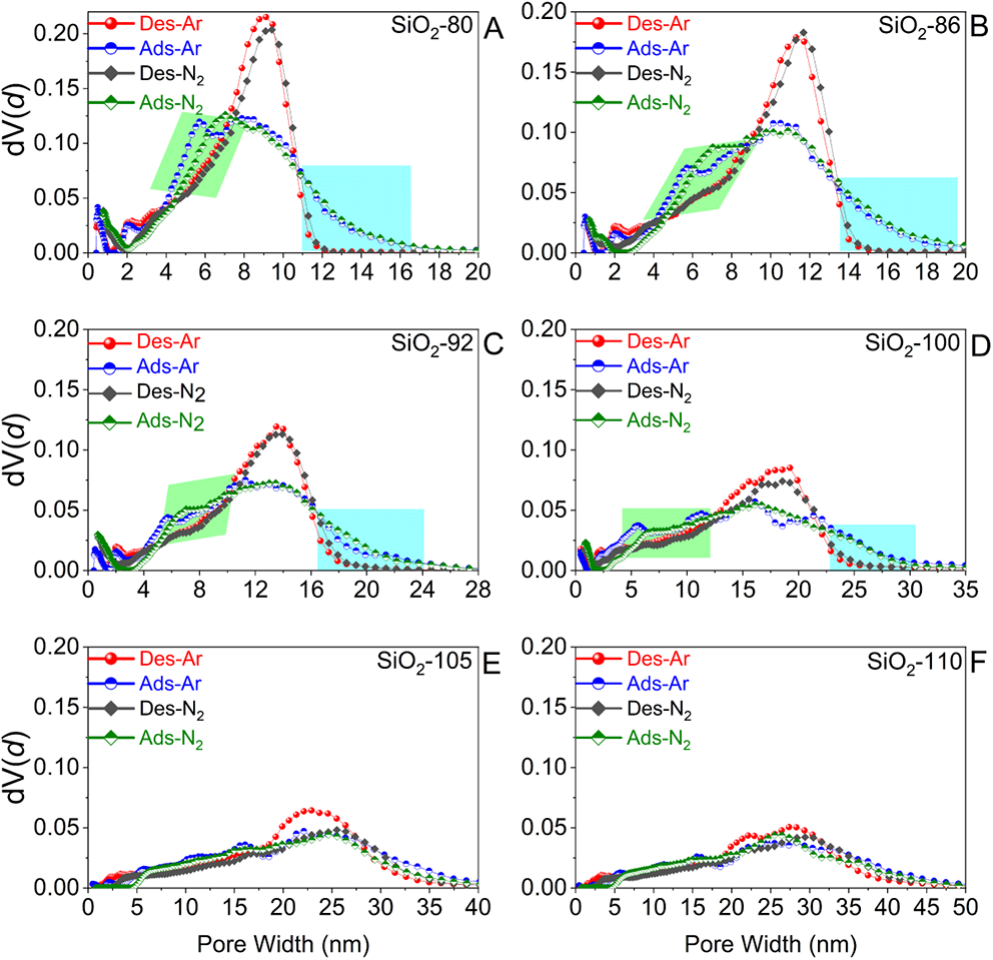
(A–F) NLDFT pore size distributions calculated from of Ar (87 K) and N_2_ (77 K) adsorption and desorption branches of physisorption isotherms of silica monoliths, as a function of the HTT. The areas displayed in green color highlight the shift of the PSD toward smaller pores calculated from adsorption isotherms, indicating the pore network effects. The bluish areas highlight those larger mesopores being connected by small-size mesopores. These larger-size mesopores thus do not appear in desorption-derived PSDs calculated from desorption branch due to pore blocking.

The PSDs determined from the adsorption and desorption branches of the Ar (87 K) and N_2_ (77 K) isotherms exhibit interesting features (Figures 6 and S5), especially as a function of the HTT:

1. Comparing the two different adsorptives, Ar (87 K) vs N_2_ (77 K), the respective desorption-based PSDs are practically identical for one particular HTT, implying the absence of cavitation as a pore emptying mechanism. Also, the adsorption-based PSDs are almost identical for the two adsorptives, suggesting that the absolute values obtained for the PSDs are reliable.^52^
2. For HTT up to 92 °C, the PSDs obtained from the adsorption branch are different from the desorption-based PSDs for both adsorptives, Ar (87 K) and N_2_ (77 K), in two features: first, the desorption-based PSDs are sharper and level off at smaller diameters (approximately 14–16 nm) than the adsorption-based PSDs (>20 nm). Second, the adsorption-based PSDs indicate a larger fraction of mesopores in the range of 4–7 nm compared to the desorption-based PSDs. Interestingly, the mode value for the average pore diameter is larger for the desorption-based analysis compared to the adsorption-based PSD. These peculiarities point to two structural properties of the mesopore space: on the one hand, it can be described as an interconnected pore network rather than independent mesopores. The filling of small connecting pores generates menisci, which may reduce the nucleation barrier for condensation in the empty pores. Thus, condensation will occur at pressures slightly below the pressure commonly predicted for delayed condensation, and the average pore diameter determined from the adsorption branch will appear slightly smaller. Note that a shift of the adsorption branch to smaller pore sizes, compared to desorption-based PSDs, has also been reported for such monoliths, which is accounted for as “initiated condensation”.^71^ Such a shift is attributed to a mismatch in the assumed connectivity of pores in the applied adsorption kernels, which describe adsorption in well-accessible and independent cylindrical pores, thereby not taking into account three-dimensional connectivity (Figure 5, case C). This effect was, for instance, described by the Kleitz group for physisorption on KIT-6 silica.^72^

On the other hand, a fraction of larger mesopores (>ca. 12 nm) is surrounded by necks of smaller diameters, resulting in the “pore-blocking” phenomenon.

For all HTTs above 92 °C, the PSDs exhibit increased pore diameters and lie almost on top of each other (Figures 6 and S5), while the overall pore dimensions increase with rising HTT. This similarity of all PSDs points to the absence of narrow connecting pores and, thus, the absence of pore blocking or cavitation.

To quantitatively assess pore network effects, the mesopore volumes were calculated from the cumulative pore volumes of all samples. Notably, comparing these distributions, calculated from NLDFT adsorption (Figure 7A) and NLDFT desorption branches (Figure 7B), reveals how variations in pore volume fractions across different mesopores’ size ranges correlated with changes in pore network connectivity. As shown in both parts of Figure 7, only a tiny volume fraction of micropores is present, which disappears above 100 °C, probably due to the merging of smaller pores. Similarly, the volume fraction of mesopores ≤5 nm decreases consistently for both adsorption and desorption branches across all samples. Additionally, the volume of 5–11 nm mesopores (blue line) declines upon increasing treatment temperature; however, the volume for this range of mesopores (5–11 nm) derived from the adsorption branch differs from those calculated from the desorption branch.

**Figure 7:**
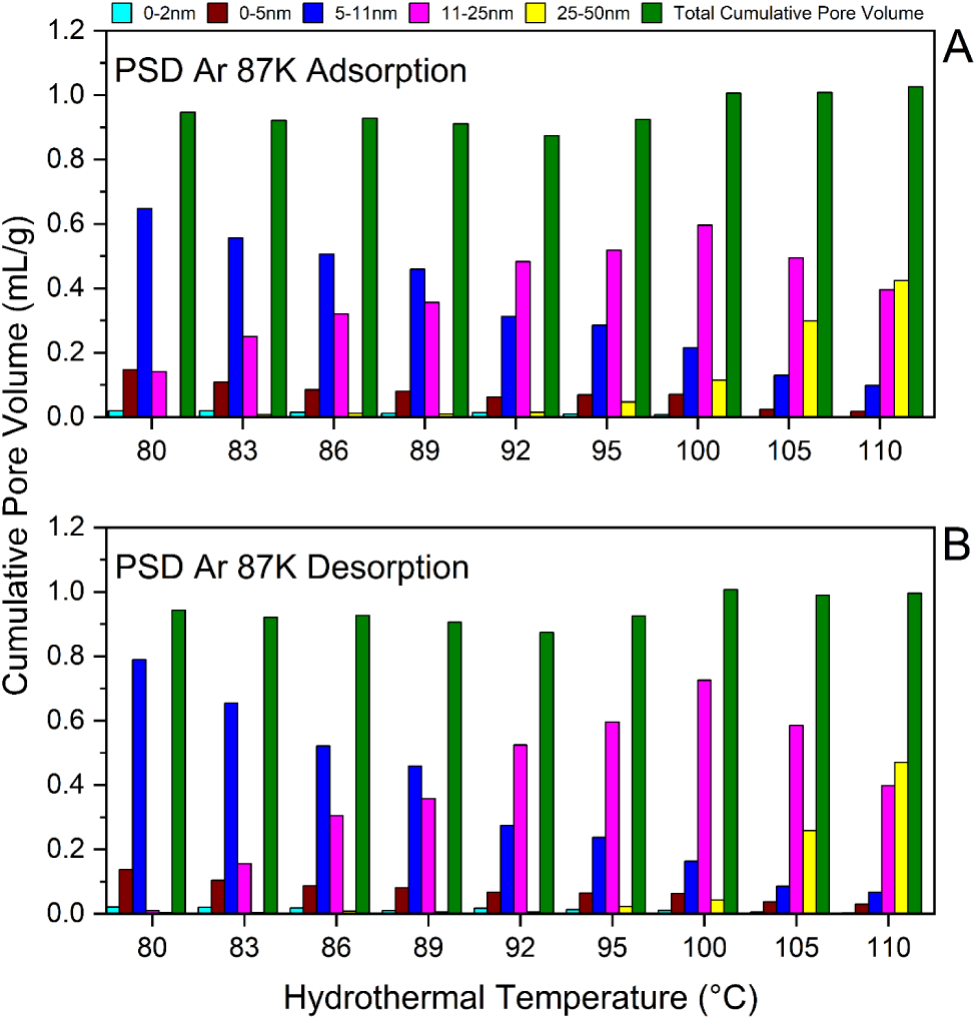
(A,B) Cumulative pore volume distributions in different ranges of mesopores, calculated by applying the NLDFT method on adsorption and desorption branches (Ar 87 K).

This shift in volume fractions of the 5–11 nm mesopores is particularly prominent in monoliths treated at 80 °C, 83 °C, and 86 °C. For instance, in the case of SiO_2_-80 the volume of 5–11 nm mesopores is almost 0.8 mL/g from desorption, compared to 0.67 mL/g for the adsorption branch. This difference (0.13 mL/g) matches the difference in pore volumes of the 11–25 nm population (pink line) as calculated from the adsorption and desorption branches. This apparent redistribution of mesopore volume from the 11–25 nm range (adsorption) to the 5–11 nm range (desorption) suggests that 0.13 mL/g of 11–25 nm mesopores are connected by necks in the 5–11 nm range, leading to pore blocking. Through this analysis, the mesopore volume fraction differences and redistribution in smaller ranges enable the determination of:

(a) which range of mesopores is restricted,
(b) the volume of restricted mesopores,
(c) and which neck sizes are responsible for pore restrictions.

Based on this interpretation, the 11–25 nm mesopores are restricted due to pore blocking rather than cavitation, as the neck sizes responsible for the restriction exceed the critical neck width *w*_*c*_ (5–7 nm) responsible for cavitation.^51,73,74^ From SiO_2_-95 onward, the emergence of 25–50 nm mesopores becomes apparent, with their contribution to total pore volume increasing progressively. By SiO_2_-100, the 11–25 nm mesopore population begins to decline, transitioning into the 25–50 nm population, which dominates the cumulative pore volume at 110 °C under extreme hydrothermal treatment conditions.

### Desorption Hysteresis Scanning for Advanced Interpretation of Pore Network Effects and Heterogeneity in the Mesoporous Space

For further understanding the pore connectivity and elucidating the type of pore network effects after hydrothermal treatments, desorption hysteresis scans were performed on specifically chosen samples, according to their distinct hysteresis loops. Argon at 87 K was used for the analysis, and four segments of desorption isotherms were recorded for each sample. In “desorption hysteresis scanning” experiments, multiple cycles of adsorption and desorption were performed: the relative pressure, at which desorption is initiated, was reduced in a stepwise fashion, and a desorption scan was measured. From the contours of these desorption branches the underlying desorption mechanism can be inferred.^54,71^ Thus, desorption starts from different filling states of the mesopores depending on the *p/p* ^o^ value. Sorption cycles were conducted at different, representative *p/p* ^o^, and the desorption mechanisms were determined using the IUPAC classification, (Figure S7; a GIF is available as animated explanation of desorption scanning mechanism in the Supporting Information), which contains three different types of hysteresis loops and pore evaporation mechanisms. Figure 8B–D shows hysteresis scans of different samples having distinct boundary loops and related desorption segments that reveal the influence of HTT on the mesopore connectivity. Hysteresis isotherms along with their complete adsorption and desorption segments are illustrated by Figure S6. The sample SiO_2_-83, treated at the lowest HTT (Figure 8B), exhibits a boundary loop with an H2­(a)-type hysteresis loop,^71^ such shape already indicating a pore network effect. All four segments of the desorption scans bend sharply and meet at the same point at lower *p/p* ^o^, revealing that evaporation is dependent on the neighboring pores. The overall evaporation from the pore network takes place by network percolation,^71^ so that all desorption segments merge together at the same lower *p/p* ^o^ value. Note that the desorption segments do not coincide at the lower closure point of the boundary loop but at a higher *p/p* ^o^. Thus, the desorption patterns are not straightforward and, importantly, cannot be attributed to one of the general types of hysteresis patterns. Recently, it was suggested that such desorption scanning features might be due to spatial mesopore size gradients within this type of silica monoliths. Such kind of effect was first reported by Meinusch et al. in 2017 postulating that the mesopores toward the outer surface of the skeleton are larger compared to the core of the skeleton.^61^ In 2020 Kube and Turke et al. used small-angle X-ray scattering (SAXS) coupled *in situ* with physisorption to further elucidate this pore size gradient effect in such silica monoliths. Their work indeed revealed a gradient in mesopore size, getting smaller while moving into the center of monolith. It was proposed that such potential spatial heterogeneity of mesopores cannot be elucidated by conventional isotherm analysis, but only scanning desorption lines expose such a phenomenon. Also, in the study of Kube et al., the scanning hysteresis lines merged into a main desorption isotherm before the closing point of the hysteresis loop, as compared to “pore blocking” effects where all the desorption scanning lines coincide at the lower closure point of the hysteresis loop.^71^ Thus, the shape of hysteresis lines reflects the pore blocking phenomenon, but additionally we propose that the merging of lines before the closure point of the hysteresis loop is influenced by spatial pore size gradient effect resulting in a mixture of hysteresis types (small hysteresis models as in Figure 8B). In sample SiO_2_-95 (Figure 8C), the boundary loop can be interpreted as a mixture of H2­(a) and H1-type hysteresis loops. The first three desorption segments bend sharply until merging together first and then meeting the boundary loop at higher *p/p* ^o^ values compared to sample SiO_2_-83. By contrast, the fourth desorption segment intersects the boundary loop independently of the other three at the lower closure point. This kind of desorption mechanism reveals that the evaporation, especially of the larger mesopores, still depends on the connecting pores (network percolation) and is, in addition, possibly affected by a gradient in the mesopore diameter, so that the first three segments evaporate at higher *p/p* ^o^ values. The sample SiO_2_-100 (Figure 8D) possesses a typical H1-type boundary hysteresis loop, revealing an independent open mesopore network. Therefore, the desorption segments merge into the boundary loop independently, which is in agreement with the conclusions drawn from the PSDs and previous studies.^55,72^ Despite these findings, the exact mechanism behind the heterogeneity in pore size distribution (PSD) across the monolith remains debated. It is unclear whether this is due to variations in pH and diffusion across the monolith’s width or if it is primarily influenced by internal vapor pressure during hydrothermal treatment.

**Figure 8:**
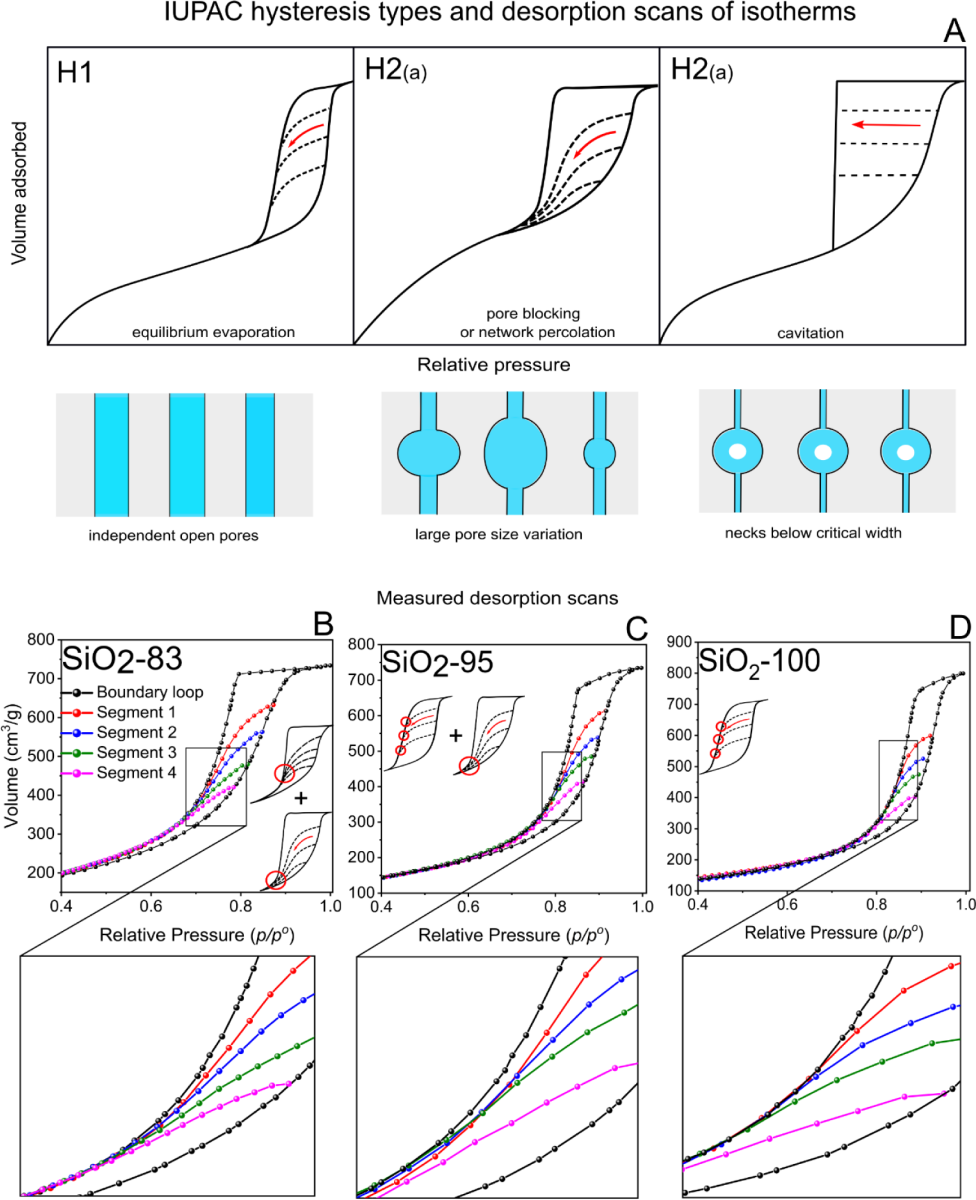
(A) Overview of IUPAC hysteresis types and related desorption hysteresis scans of mesoporous materials with different pore network. Reproduced from Kube and Turke et al. Copyright 2020 American Chemical Society. (B–D) Hysteresis loops of three samples treated at different HTTs having four desorption scanning isotherms each (Ar 87K). (B) (SiO_2_-83) exhibits a H2­(a) type hysteresis loop, C (SiO_2_-95) exhibits a mixture of H2­(a) and H1, while (D) (SiO_2_-100) reflects the H1 hysteresis loop. For visual clearance, the hysteresis lines are zoomed-in in separate graphs. The insets in (B), (C), and (D) illustrate the fundamental types of IUPAC hysteresis loops and desorption scanning lines.

Thus, based on desorption hysteresis scans, the following additional insights into the evaporation mechanisms of different pore networks can be extracted:

(i) The shape of the boundary loops does not allow for unambiguously determining the evaporation mechanism, but desorption scanning analysis provides further insight.
(ii) Evidence of network percolation can be deduced from desorption hysteresis lines if all lines converge at the same *p/p* ^*o*^ value.
(iii) Desorption scans help to possibly identify spatial heterogeneity in PSD’s or spatial pore size gradient effects.^71^

Based on the interpretation that the merging of desorption scanning isotherms at higher values of the hysteresis loop reflects the heterogeneity of the pore network, the evaporation mechanism in such a porous system is illustrated in Figure 9a. This translates the experimental data into a model for a possible pore network present in sample SiO_2_-83 that illustrates the spatial pore size gradient effects as suggested by the desorption scanning isotherms in Figure 8B. In the underlying model, all the pores are connected to their neighboring pores in three dimensions. The mean pore size decreases toward the core of the monolith, while the largest mesopores open into the macropore space. The main desorption isotherm in Figure 9b evaporates in the sequence A–B1–C–D (Figure 9c), where at point A, all the pores are filled, and at point D, the hysteresis loop closes. In comparison, the desorption scanning isotherm (Figure 9b) starts at point B2, where the majority of larger mesopores have already emptied, and the pore size gradient creates three regions within the pore network: i.e. (i) the dense core (smallest pores) with all the filled pores, (ii) the semi-dense shell farther outward having only a fraction of filled pores, and (iii) the larger pores toward the outer surface that are empty. This desorption scanning branch merges into the main isotherm at point C, where all the pores from the semi dense regions are vacant. The pores evaporate by both external and internal evaporation depending on the state of neighboring pores. At point C, the empty state of the pores is identical to the main desorption isotherm, thus the scanning desorption isotherm merges at this point. From point C–D, the entire pore network continues to empty, so the pores only evaporate into larger mesopores and then into external space (macropores) until point D, where all the mesopores are empty. A possible schematic for the open mesopore network of sample SiO_2_-100 is presented in Figure 9d as a reflection of the desorption scanning isotherms from Figure 8D.

**Figure 9:**
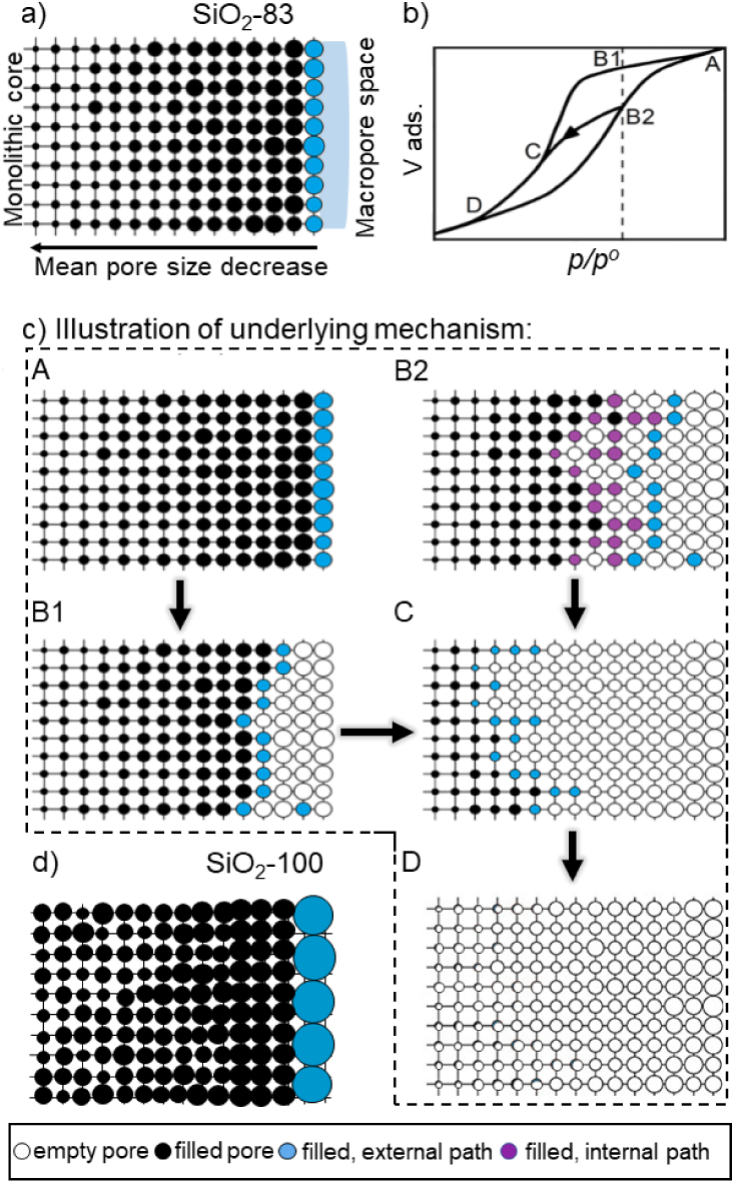
Anticipated model for the spatial heterogeneity of the mesopore space in silica monoliths in the form of a pore size gradient. Reproduced from Kube and Turke et al.^71^ (copyright 2020 American Chemical Society). (a) A schematic illustrating the mesopore space in SiO_2_-83 with heterogeneously distributed mesopores; (b) isotherm and desorption hysteresis line for a pore network that exhibits a pore size gradient; (c) illustration of the mechanism of evaporation according to the isotherm and hysteresis line in **Figure 9b**, (d) model illustrating the large-size mesopore space in sample SiO_2_-100: in spite of a possible gradient, the mesopores can be regarded as independent pores in terms of the physisorption behavior.

## Conclusions

This study aimed to elucidate details in the synthesis of meso-macroporous SiO_2_ monoliths generated by the widespread synthetic strategy established by Nakanishi et al. These materials are distributed as commercial products and are routinely used as stationary phases in liquid chromatography, especially in the pharmaceutical industry and research. Their usage is based on a well-defined and optimized macropore and mesopore space, which combines excellent flow and separation properties. However, the underlying synthetic steps are delicate, possibly impeding reproducible synthesis. In general, the synthetic process is quite sensitive to slight changes in temperature during the two relevant steps in the synthesis: within the initial gelation step, spinodal decomposition occurs between the siliceous phase and the polymer-containing phases, which ultimately creates the macropores (Figure 1). In our previous publication, we showed that this step is quite sensitive even to small temperature variations on the order of 1 K, allowing for tuning the macropore dimension.^58^

Here, we focus on the subsequent harsh hydrothermal treatment step (Figures 1 and 10), which involves the release of NH_3,aq_ from urea, and the basic environment widens small cavities (micropores) into the desired mesoporous network. While this synthesis is well established and yields materials used as commercial stationary phases in HPLC, here we attempted to examine the relationship between the hydrothermal treatment step and the concomitant final mesopore space.

**Figure 10:**
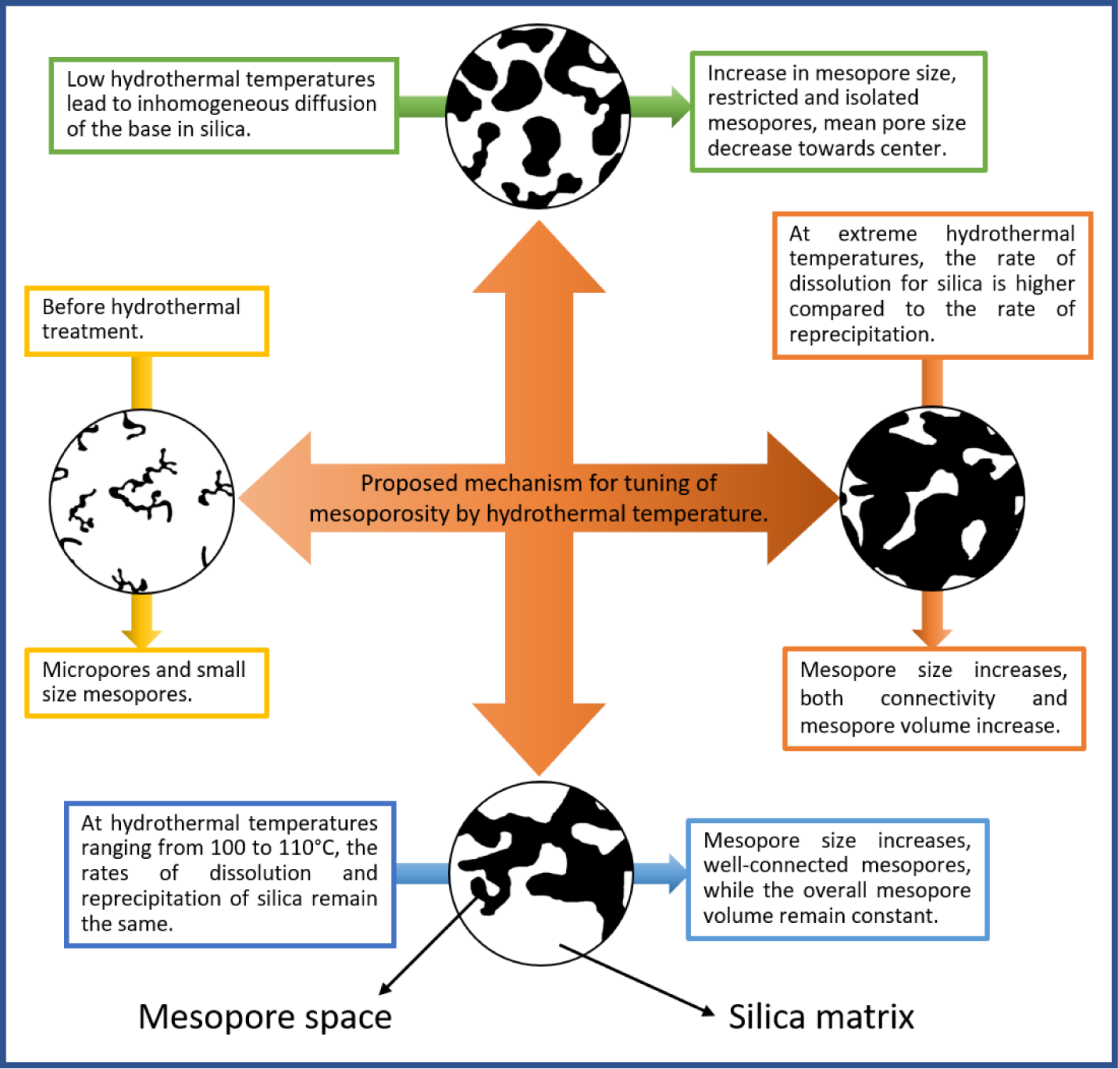
Proposed mechanism of hydrothermal treatment that controls the size of mesopores, pore network effects, and the spatial gradient in pore size distribution across the width of silica monoliths by changing the rate of silica dissolution of silica by base diffusion.

In the synthesis, tuning of the mesoporosity is typically achieved by applying different hydrothermal temperatures. Here, we synthesized SiO_2_ monoliths by applying HTT between 80 and 110 °C, which resulted in a systematic change in the mesopore dimension and the connectivity between the mesopores. State-of-the-art physisorption analysis, using Ar (87 K) and N_2_ (77 K) as adsorptives, revealed that a 3 °C increment in HTT (from 83 to 95 °C) led to an approximately 1 nm increase in the average mesopore diameter, ranging from 11 to 15 nm. Thus, modifying the HTT enables a precise variation of the mesopore dimension. In the range of 95 to 110 °C, with every 5 °C change in HTT, a more pronounced increase of 4 nm is observed, yielding tunable average mesopore sizes from 15 to 27 nm. The influence of the base treatment and HTT on the mesopore space is summarized in Figure 10.

The most relevant insight of this study was obtained regarding the connection between the mesopores, which is a significant structural feature with respect to the separation properties in HPLC. Possible hindrance in the mutual connection, e.g., in the form of bottlenecks (see Figure 8), can potentially impede diffusion in the mesopore space. Thus, desorption hysteresis scanning analysis was performed on selectively chosen samples and revealed the evaporation mechanism and, consequently, the connection within the mesoporous network. The desorption scanning isotherms of samples SiO_2_-83 and SiO_2_-95 with type H2­(a) hysteresis revealed pore blocking. Additionally, we interpret the merging of hysteresis lines at higher *p/p* ^o^ as an indication of the presence of spatial pore size gradient effects within the silica monoliths, confirming recent *in situ* physisorption SAXS studies. However, with an increase in HTT to finally 100 °C, the mesopore size distribution broadened, accompanied by the evolution of a well-connected mesoporous network without hindrance in connection. Despite these findings, the exact mechanism behind the heterogeneity in pore size distribution (PSD) across the monolith remains debated. It is unclear whether this is due to variations in pH and diffusion across the monolith’s width or if it is primarily influenced by internal vapor pressure during hydrothermal treatment.

From our analyses, we conclude that within the hydrothermal treatment, the rate of diffusion of the base within the material not only controls the size of mesopores but also affects the spatial distribution of mesopores of different sizes. Lower HTT involves a smaller concentration of OH^–^ ions and may not be sufficient to overcome energetic barriers. This may result in a spatial pore size gradient across the diameter of a strand, confirming recent *in situ* physisorption SAXS studies on monoliths.^68^

The previous studies on hydrothermal treatment for tuning mesoporosity in silica monoliths focused mainly on the application perspective, particularly in HPLC.^43–50^ Our present study complements these studies and contributes to a better understanding of the relevant hydrothermal synthetic step. The insights might allow for advanced control of the mesopore size and pore network connectivity effects via hydrothermal treatment, which could help improve the properties of these monoliths for applications in separation and heterogeneous catalysis.

## Supplementary Material



## Notes

Figure S1: Nitrogen and argon isotherms for all samples; Figure S2: Comparison of PSD using different kernels; Figure S3: Isotherms and PSD for SiO_2_-100-1 and SiO_2_-100; Figure S4: Total cumulative surface area and cumulative pore volume; Figure S5: PSD for samples SiO_2_–-83, 89, and 95; Figure S6: Complete cycles of adsorption hysteresis scanning; Figure S7: GIF related to the mechanism of desorption hysteresis scanning (PDF)
