# Supplementary material for: Tuning the Mesopore Network in Meso-Macroporous Silica Monoliths by Hydrothermal Treatment – A Physisorption Study
Source: Langmuir. 2025 May 28;41(22):13845–59. doi: 10.1021/acs.langmuir.5c00572 (PMC12164339; doi:10.1021/acs.langmuir.5c00572)
Supplement: Supplementary file 1 [file la5c00572_si_001.pdf]

Tuning the Mesopore Network in Meso-Macroporous Silica Monoliths by Hydrothermal Treatment – a Physisorption Study

Usman Ali[a,b], Rafael Meinusch[a,b], Kevin Turke[a,b], Peter R. Schreiner[b,c], Bernd M. Smarsly[a,b]*

a. Institute of Physical Chemistry, Justus Liebig University Giessen, D-35392 Giessen, Germany

b. Center for Materials Research, D-35392 Giessen, Germany

c. Institute of Organic Chemistry, Justus Liebig University Giessen, D-35392 Giessen, Germany

Supporting Information

Table of figures

Figure S 1.A-D Physisorption isotherms for silica monoliths measured by two different adsorptives (Ar at 87 K and N_2 at 77 K)……………………………………………………3

Figure S 2.AB Comparison of PSDs calculated from NLDFT cylindrical kernel for Argon 87 K and Nitrogen 77 K with NLDFT cylindrical and spherical kernel for Argon 87 K and Nitrogen 77 K. 4

Figure S 3. (A) Ar 87 K physisorption isotherms for two silica monolithic samples treated at the same hydrothermal temperature but different treatment time: SiO_2-100 for 28 hours and SiO_2-100-1 for 20 hours. (B) Comparison of PSD calculated from NLDFT desorption isotherms for silica. Both figures indicate that different hydrothermal treatment times result in no significant difference in the isotherms and pore size distribution (PSD). However, the cumulative pore volume of SiO_2-100 is slightly higher than that of SiO_2-100-1 due to the longer hydrothermal treatment time. SiO_2-100 underwent a total of 28 hours of treatment (13 hours heating ramp plus 15 hours holding), while SiO_2-100-1 was treated for 20 hours (10 hours heating ramp plus 10 hours holding). 4

Figure S 4. A and B show the cumulative surface area and cumulative pore volume distributions contributed by the range of mesopores size and pores larger than 50 nm. 5

Figure S 5.A-C Pore size distributions calculated from Ar (87 K) and N_2 (77 K) adsorption and desorption branches of physisorption isotherms using NLDFT method for cylindrical pores.................6

Figure S 6. Hysteresis isotherms along with their complete adsorption and desorption segments showing the complete mechanism of hysteresis scanning measured with Ar (T = 87 K). 8

Figure S 7. An animated representation of desorption hysteresis scanning mechanism that shows how experiment in multiple cycles of adsorption and desorption is performed. The relative pressure, at which desorption is initiated, is reduced in a stepwise fashion, and a desorption scan is measured. The desorption starts from different filling states of mesopores depending on the p/p^o value. Sorption cycles were conducted at different, representative p/p^o and the desorption mechanisms determined using the classification of IUPAC. 9

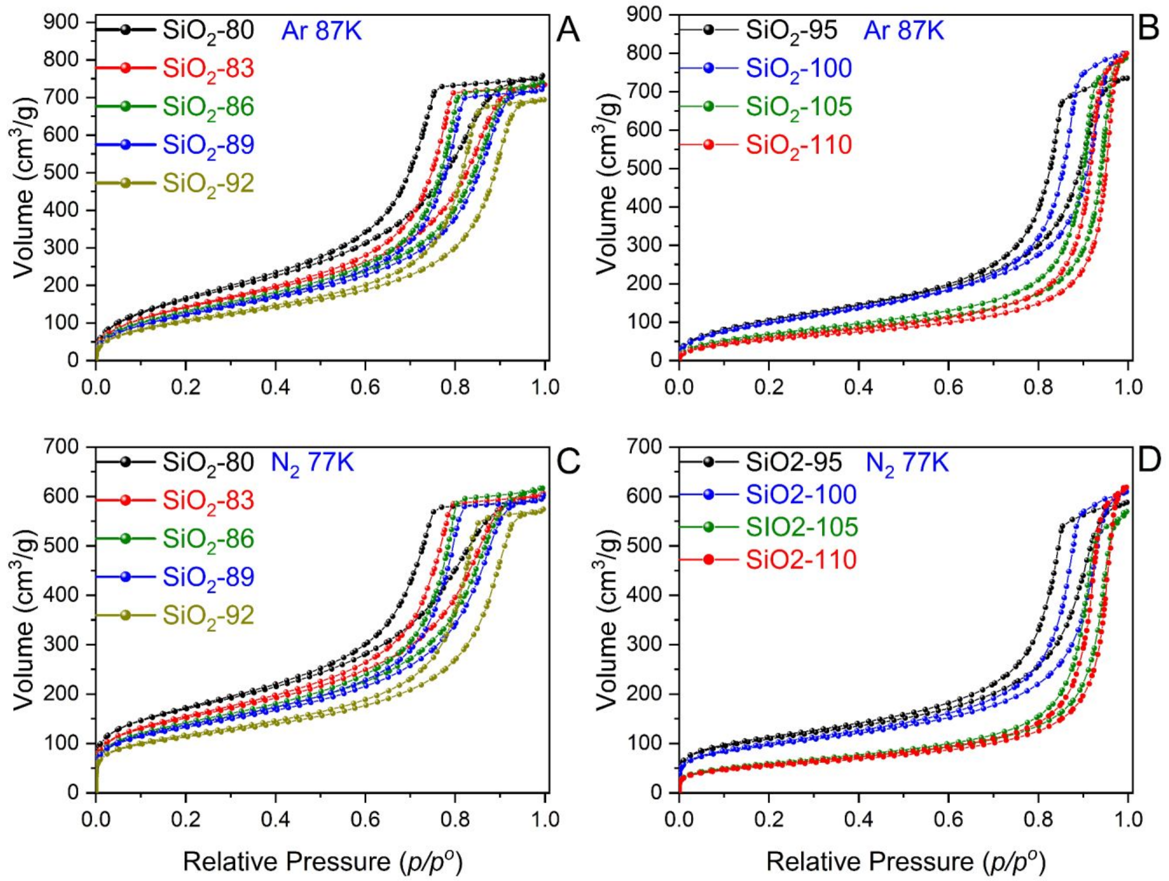

Figure S 1. A-D Physisorption isotherms for silica monoliths measured by two different adsorptives (Ar at 87 K and N_2 at 77 K).

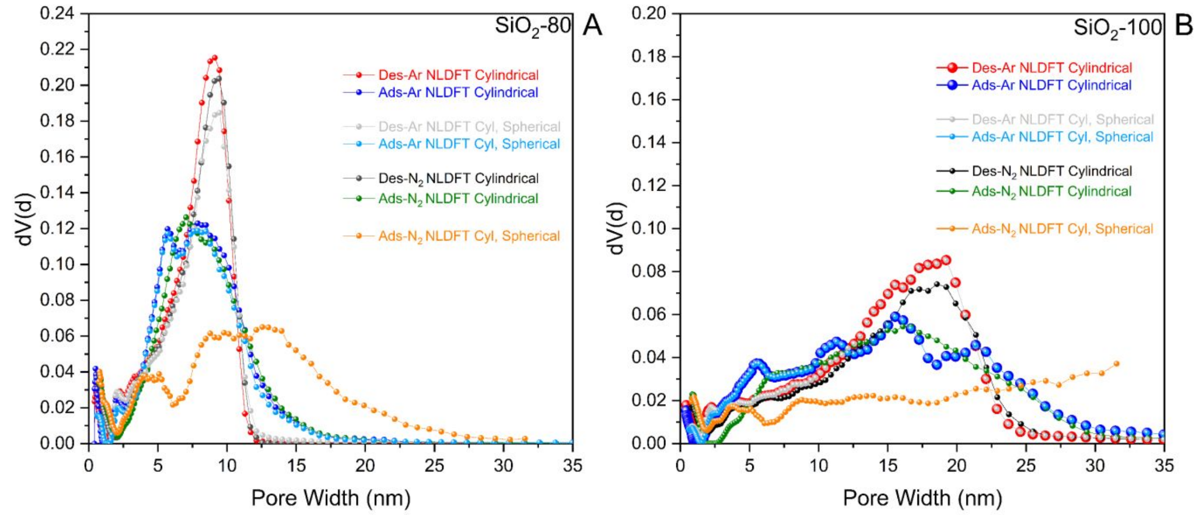

Figure S 2. AB Comparison of PSDs calculated from NLDFT cylindrical kernel for Argon 87 K and Nitrogen 77 K with NLDFT cylindrical and spherical kernel for Argon 87 K and Nitrogen 77 K.

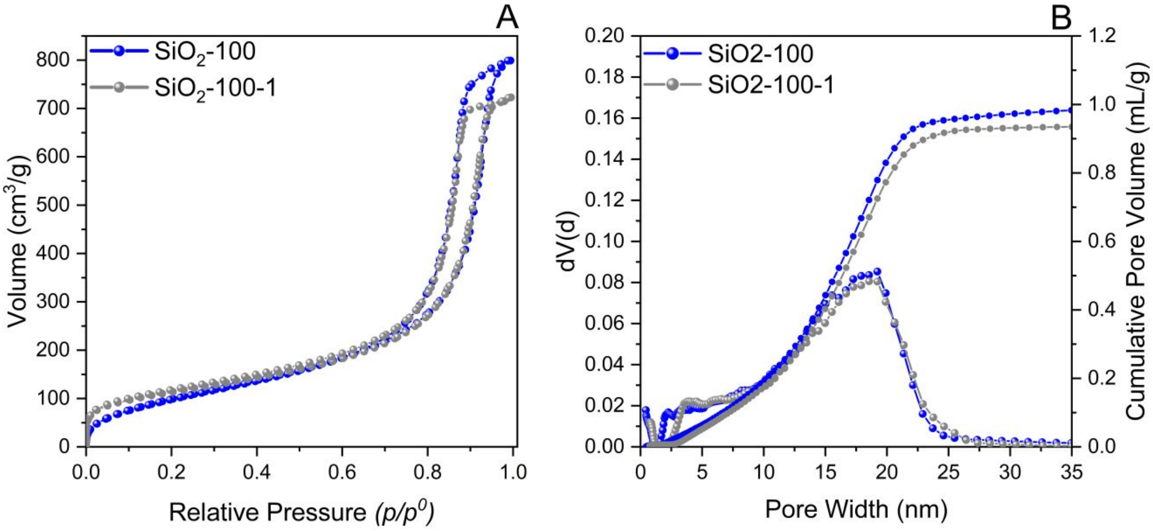

Figure S 3. (A) Ar 87 K physisorption isotherms for two silica monolithic samples treated at the same hydrothermal temperature but different treatment time: SiO_2-100 for 28 hours and SiO_2-100-1 for 20 hours. **(B)** Comparison of PSD calculated from NLDFT desorption isotherms for silica. Both figures indicate that different hydrothermal treatment times result in no significant difference in the isotherms and pore size distribution (PSD). However, the cumulative pore volume of SiO_2-100 is slightly higher than that of SiO_2-100-1 due to the longer hydrothermal treatment time. SiO_2-100 underwent a total of 28 hours of treatment (13 hours heating ramp plus 15 hours holding), while SiO_2-100-1 was treated for 20 hours (10 hours heating ramp plus 10 hours holding).

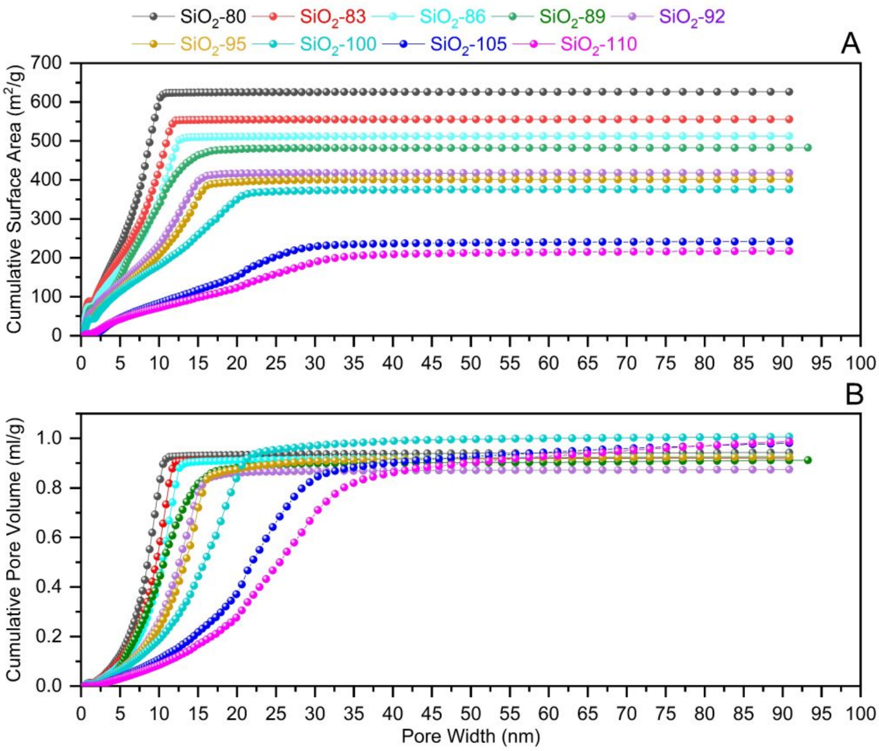

Figure S 4. **A** and **B** show the cumulative surface area and cumulative pore volume distributions contributed by the range of mesopores size and pores larger than 50 nm.

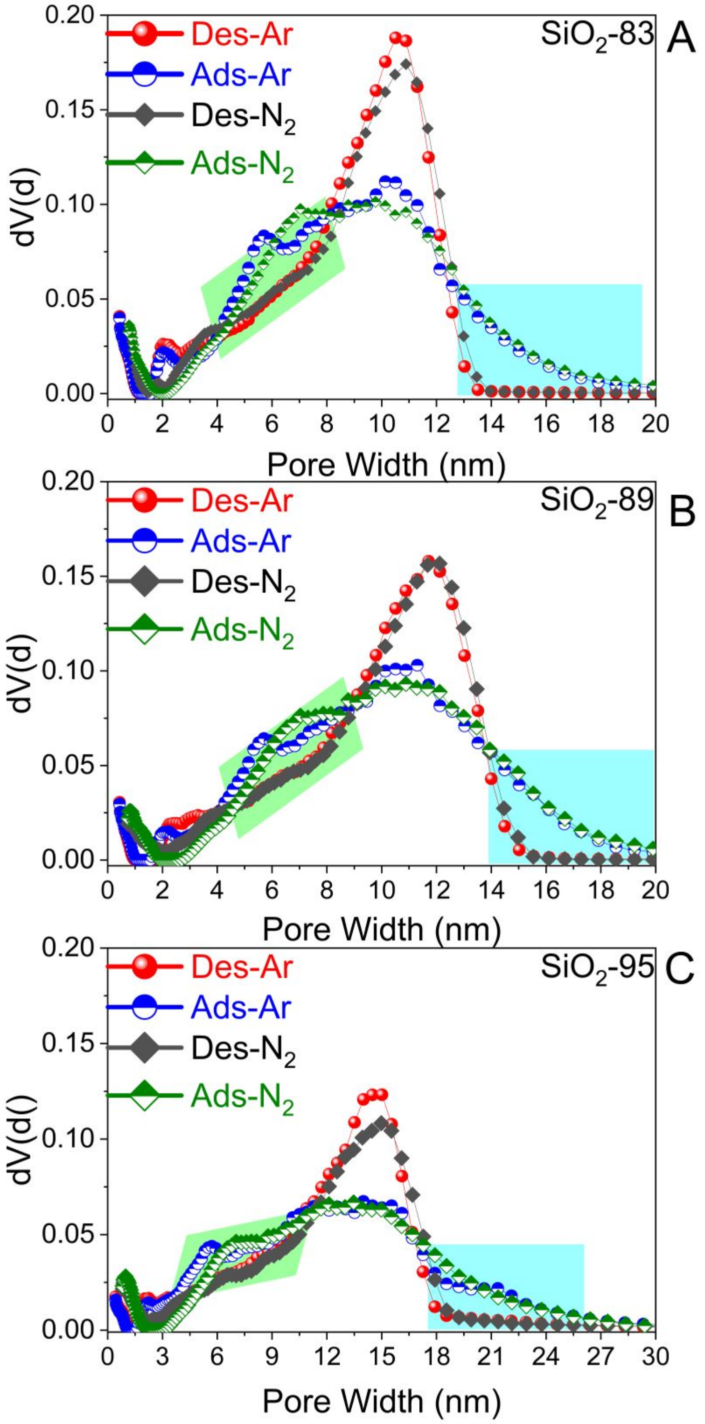

Figure S 5. A-C Pore size distributions calculated from Ar (87 K) and N_2 (77 K) adsorption and desorption branches of physisorption isotherms using NLDFT method for cylindrical pores.

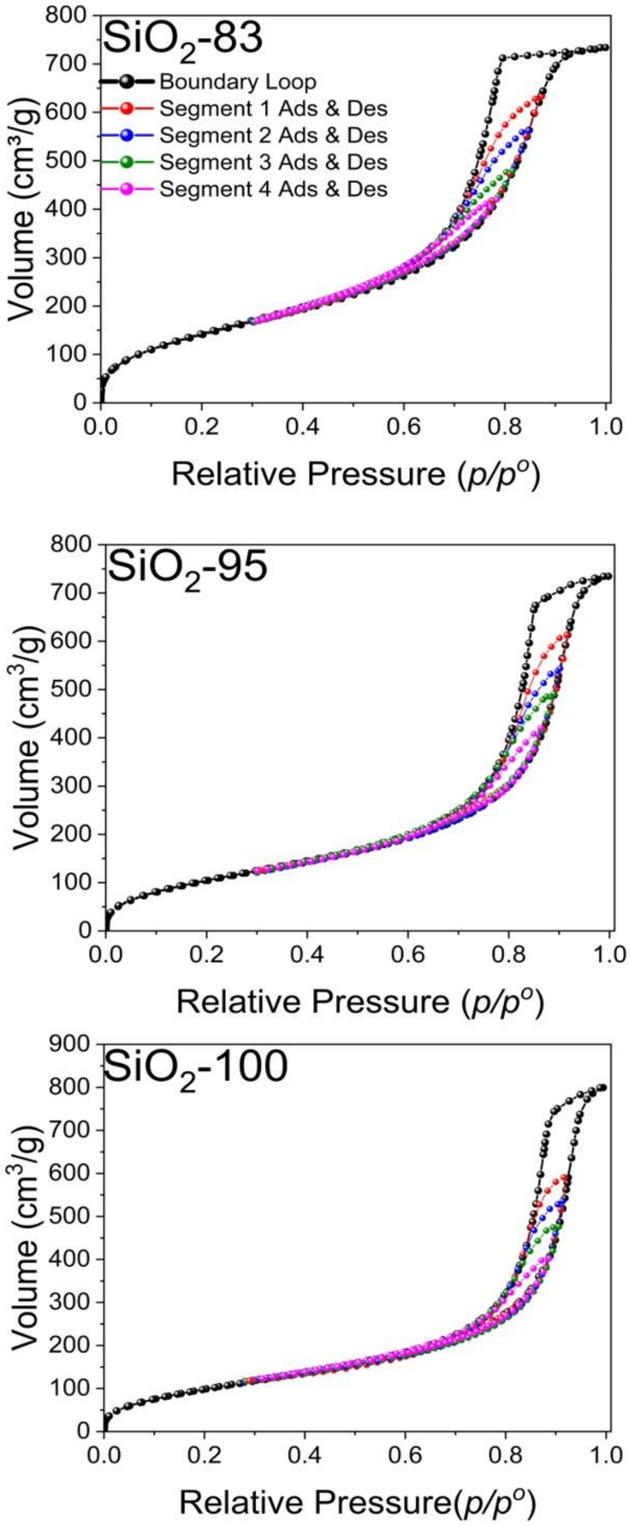

Figure S 6. Hysteresis isotherms along with their complete adsorption and desorption segments showing the complete mechanism of hysteresis scanning measured with Ar ($T = 87$ K).

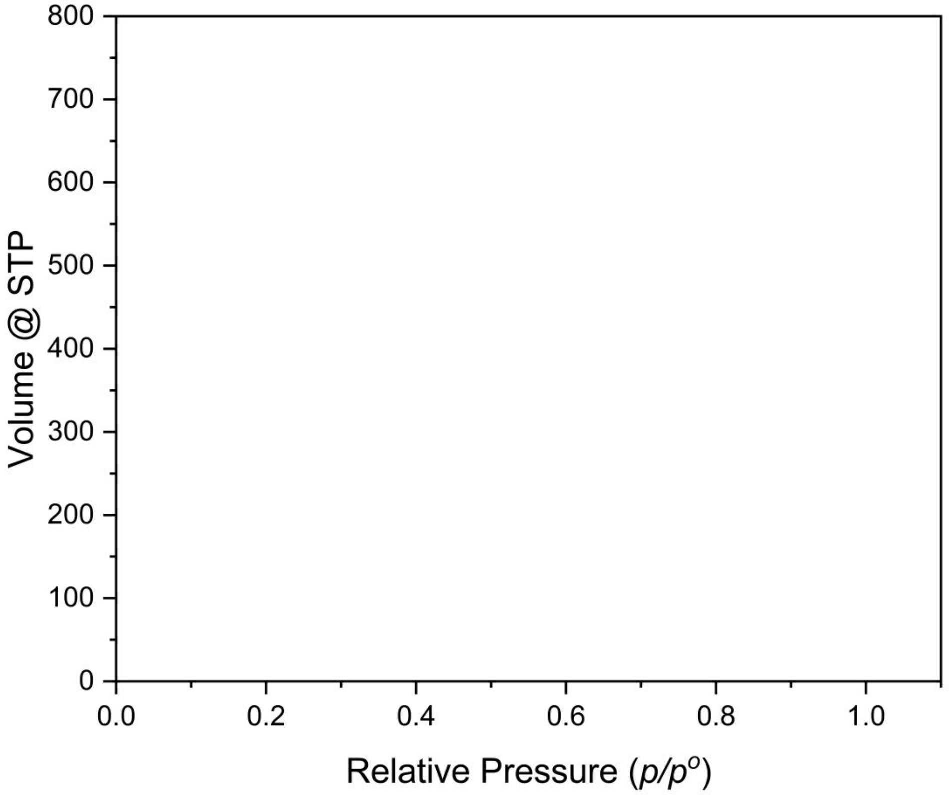

Figure S 7. An animated representation of desorption hysteresis scanning mechanism that shows how experiment in multiple cycles of adsorption and desorption is performed. The relative pressure, at which desorption is initiated, is reduced in a stepwise fashion, and a desorption scan is measured. The desorption starts from different filling states of mesopores depending on the p/p^o value. Sorption cycles were conducted at different, representative p/p^o and the desorption mechanisms determined using the classification of IUPAC.
